# Supplementary material for: Vitamin D Modified DSS-Induced Colitis in Mice via STING Signaling Pathway
Source: Biology (Basel). 2025 Jun 18;14(6):715. doi: 10.3390/biology14060715 (PMC12190092; doi:10.3390/biology14060715)
Supplement: Supplementary file 1 [file biology-14-00715-s001.zip › Supplementary Materials S1: 16s-report of gut microbiota/Groups/diff_analysis/RF/Species_Groups.RFimportance.pdf]

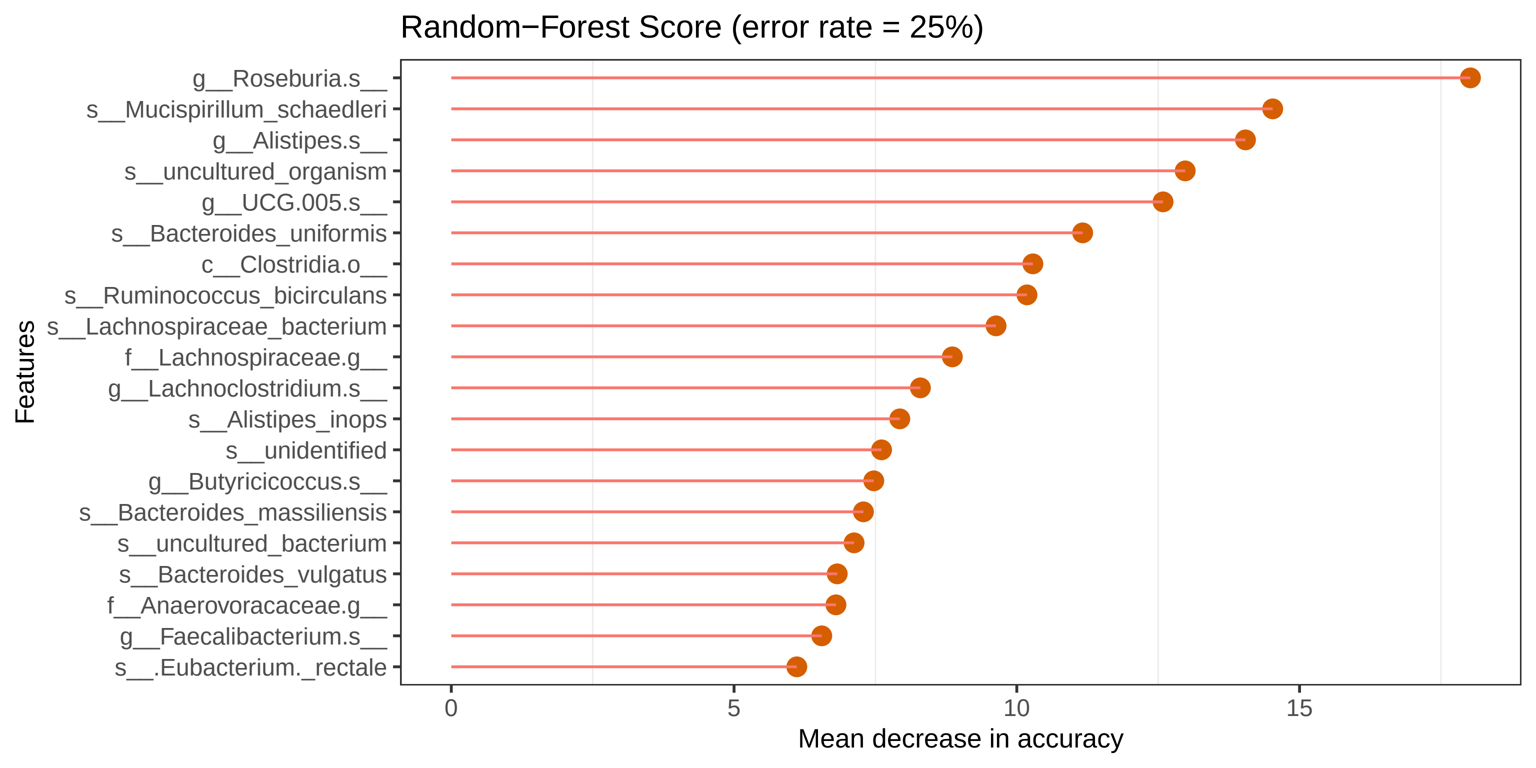
Random−Forest Score (error rate = 25%)
g__Roseburia.s__
s__Mucispirillum_schaedleri
g__Alistipes.s__
s__uncultured_organism
g__UCG.005.s__
s__Bacteroides_uniformis
c__Clostridia.o__
s__Ruminococcus_bicirculans
s__Lachnospiraceae_bacterium
f__Lachnospiraceae.g__
g__Lachnoclostridium.s__
s__Alistipes_inops
s__unidentified
g__Butyricicoccus.s__
s__Bacteroides_massiliensis
s__uncultured_bacterium
s__Bacteroides_vulgatus
f__Anaerovoracaceae.g__
g__Faecalibacterium.s__
s__.Eubacterium._rectale
Features
0
5
10
15
Mean decrease in accuracy
